# The impact of public hospital performance-based pay scheme reform on revenue structure and service efficiency under the Diagnosis-Intervention Packet payment system: evidence from a pilot city in China

**DOI:** 10.1186/s12913-026-14449-7

**Published:** 2026-03-28

**Authors:** Huanyu Shi, Baoli Su, Kun Wang, Dian Zhang, Zhichao Liu, Yang Zhang, Zhichao Cheng

**Affiliations:** 1 School of Economics and Management, Beihang University, Beijing, 100191 China; 2 School of Medical Management, Shandong First Medical University, Tai’an, 271016 China; 3 The Second Affiliated Hospital of Shandong First Medical University, Tai’an, 271000 China; 4 Tai’an Healthcare Security Administration, Tai’an, 271000 China

**Keywords:** Case-based payment system, Diagnosis-Intervention Package, Performance-based pay scheme, Revenue structure, Service efficiency

## Abstract

**Background:**

As China transitions from fee-for-service (FFS) to case-based payment systems, the Diagnosis-Intervention Packet (DIP) system has emerged as a key approach to enhance cost containment and service efficiency. Aligning internal incentives, such as through performance-based pay schemes (PBPS), is crucial to fully realizing the intended effects of DIP.

**Methods:**

This study assessed the impact of implementing a DIP-aligned PBPS reform on hospital revenue structure and service efficiency. Using administrative data from a pilot city, we conducted controlled interrupted time series analyses, comparing public hospitals that adopted the DIP-aligned PBPS with those that retained the traditional PBPS, stratified by insurance type: Urban Employee Basic Medical Insurance (UEBMI) and Urban Resident Basic Medical Insurance (URBMI).

**Results:**

After the reform, the intervention group showed a significant monthly decline in the proportion of drug sales in inpatient expenses (− 0.76%, *P* = 0.046), particularly among UEBMI patients (− 0.875%, *P* = 0.035), with no significant change for URBMI. The proportion of laboratory tests and examinations in inpatient expenses dropped immediately in the overall sample (− 3.479%, *P* = 0.006), UEBMI (− 4.246%, *P* = 0.006), and URBMI (− 2.922%, *P* = 0.025), but subsequently showed upward monthly trends (overall: 0.495%, *P* = 0.009; UEBMI: 0.503%, *P* = 0.014; URBMI: 0.461%, *P* = 0.016). The cost consumption index declined steadily over time (overall: −0.026, *P* = 0.025; UEBMI: −0.026, *P* = 0.040; URBMI: −0.026, *P* = 0.027), as did the time consumption index (overall: −0.012, *P* = 0.032; UEBMI: −0.015, *P* = 0.036; URBMI: −0.011, *P* = 0.045).

**Conclusion:**

Integrating PBPS reform with DIP payment implementation effectively reduced drug expenditures and enhanced service efficiency in public hospitals. However, the observed differences between the UEBMI and URBMI groups raise essential concerns about equity in health service delivery, and the increase in expenses for laboratory tests and examinations suggests potential gaming behavior in response to new incentives. These findings underscore the need for continuous policy monitoring and adaptive regulation to mitigate unintended effects and ensure the success of PBPS reforms.

**Supplementary Information:**

The online version contains supplementary material available at 10.1186/s12913-026-14449-7.

## Introduction

Fee-for-service (FFS) is a traditional medical insurance payment system widely adopted due to its simplicity and transparency in administration. Implementing FFS can help hospitals reduce administrative costs related to service billing. It also enables patients to clearly understand and track itemized charges [1, 2]. However, FFS is often associated with unnecessary medical practices, inefficiencies in healthcare delivery, and uncontrolled medical expenditures, as it incentivizes physicians to provide excessive services to maximize their income [3, 4]. These inefficiencies increase the overall burden on healthcare systems and worsen disparities in access to affordable, essential care [5].

Since the 1980s, countries worldwide have implemented various payment systems to restructure the incentive mechanisms for hospitals, with case-based payment systems, such as Diagnosis-Related Groups (DRGs), receiving widespread attention [6]. The United States was the first country to implement DRGs at scale through the Medicare program in 1983 [7]. Subsequently, other countries, including Australia, Germany, and Sweden, have adopted DRGs-based systems, adapting them to the specifics of their healthcare systems [8]. Existing studies indicate that DRGs can effectively categorize inpatient services, contain costs, and improve service efficiency [9, 10]. These case-based payment systems are therefore not only financing tools, but also policy levers intended to support the redesign of hospital-based care toward greater cost-effectiveness.

In the 21st century, the Chinese government began implementing case-based payment systems nationwide to replace the traditional FFS system. After 2010, several cities, including Beijing, introduced the DRGs payment system, achieving modest success in reducing total expenditures and out-of-pocket payments [11, 12]. However, DRGs requires comprehensive clinical data and complex grouping algorithms based on robust medical evidence, posing technical and infrastructural challenges for nationwide rollout in China’s current healthcare setting [13]. A mid-term evaluation conducted in 2020 across 30 DRGs pilot cities revealed that only eight met the minimum technical standards, underscoring the system’s limited readiness for full-scale adoption [14].

To accelerate the implementation of case-based payment systems, China developed an innovative payment system known as the “Diagnosis-Intervention Packet” (DIP) [15]. Conceptually similar to DRGs, DIP operates as a fixed-payment system where hospitals are reimbursed a predetermined amount per case. However, distinct from DRGs, which rely on complex clinical algorithms, DIP employs a big-data approach to categorize cases based on specific combinations of diagnoses and medical interventions, making it more scalable in regions with varying technological readiness [16]. In practice, this big-data–driven design makes DIP substantially easier to code and implement in county-level and municipal public hospitals, where clinical information systems and documentation standards are still developing. More importantly, the DIP system aims to promote equitable healthcare access by expanding reform coverage to resource-constrained areas and underserved populations [17]. At the same time, by combining fixed case-based payments with partial financial risk transfer to hospitals, the DIP payment system is explicitly intended to encourage hospitals to redesign care processes and deliver more cost-effective inpatient services.

**Fig. 1:**
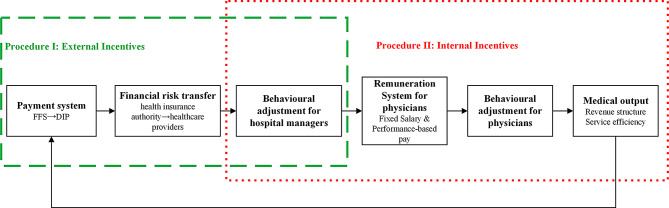
Mechanism of the impact of the payment system on medical output

The payment system is designed to serve as a powerful economic lever to influence hospitals’ behavior and service outcomes [18]. The mechanism by which the payment system influences medical output is illustrated in Fig. 1 and is divided into two stages: Process I—External Incentives and Process II—Internal Incentives. In this framework, the DIP payment system represents the external incentive environment, as it is designed and administered by the Healthcare Security Administration and applies uniformly to all hospitals within the region. Through partial financial risk transfer and point-based reimbursement, DIP alters hospitals’ budget constraints and exposes them to stronger financial accountability compared to FFS [19, 20].

Financial risk exposure influences hospital decision-making, particularly at the managerial level [21]. Hospital managers act as intermediaries, and their behavioral adjustments lead to a reform in the remuneration system, effectively converting external incentives into internal motivations for clinical staff [22]. In China, healthcare worker compensation consists of a base salary and performance-based pay [23]. While base salary provides income stability [24], performance-based pay differentiates earnings based on individual contributions, aiming to enhance staff motivation and organizational alignment [25]. Unlike DIP, which is standardized at the regional level, performance-based pay schemes (PBPS) are independently designed and implemented by each hospital. Therefore, even under the same external DIP payment environment, hospitals may vary substantially in the extent to which internal incentives are aligned with case-based payment objectives. Consequently, hospital managers can shape physician behavior under the payment system by modifying PBPS. These behavioral changes for physicians ultimately affect medical output [26, 27]. Policymakers subsequently adjust the design of the payment system based on whether the medical output meets the intended policy objectives.

The effectiveness of any payment system depends on hospitals’ perception of financial pressure and responsiveness to incentives—a process requiring synergy between external and internal incentive structures [28]. However, existing studies on DIP have primarily focused on external incentives, with limited attention on internal incentives. In practice, although DIP has been widely adopted in pilot cities, many hospital managers have not aligned their internal incentive systems accordingly. Due to policy misinterpretation or limited management capacity, some continue to follow the outdated “more work, more pay” principle, thereby weakening the intended reform effects [29, 30].

One of the primary goals of the DIP payment system is to incentivize cost-effective care through fixed payments, thereby shifting hospital revenue composition away from drug and laboratory testing expenditures toward therapeutic services and enhancing overall operational efficiency [31]. Under the FFS system, hospitals’ revenue heavily depends on the sale of drugs and the provision of laboratory tests and examinations, which fosters a profit-driven behavior in public hospitals and increases the financial burden on patients. In addition, inefficient care patterns—such as extended lengths of stay (LOS)—contribute to the overutilization of limited healthcare resources [32–34]. These issues strain the sustainability of public insurance funds and limit the system’s capacity to serve a broader population base [35]. By introducing standardized payments and shifting financial accountability to hospitals, the DIP system seeks to encourage more cost-conscious and efficient care delivery [36, 37].

This study investigates the impact of PBPS reform under the DIP payment system on revenue structure and service efficiency in Chinese public hospitals. Using data from a pilot city implementing DIP, we employed controlled interrupted time series (CITS) analyses to assess the changes in outcomes for hospitals that modified their PBPS to align with the DIP payment system compared to those that continued using traditional PBPS, both before and after the intervention.

## Institutional backgrounds

DIP is a data-driven payment system based on case classification and value-based calculations [38]. It is important to note that DIP is designed specifically for inpatient hospital services rather than for all types of healthcare expenditure. The DIP payment system operates in the following steps: The first step is case grouping. The two dimensions for grouping are diagnosis and the procedures performed. The primary diagnosis and primary procedures (including surgical and therapeutic interventions) are identified using the International Classification of Diseases, Tenth Revision (ICD-10), and the International Classification of Diseases, Ninth Revision, Clinical Modification (ICD-9-CM3), respectively. Preset programs automatically complete this process without manual intervention. The second step is determining the points for each group. The points for each group are based on the resource consumption weight, which is calculated by dividing the average hospitalization expenditures of cases within a given DIP group by the overall average hospitalization expenditures across all cases. A higher point indicates higher resource consumption for the group, which results in higher compensation for the hospital. The third step is determining the point value (PV). The PV represents the actual value of each point, which is calculated by dividing the total medical insurance funds used for the DIP payment in the region by the total points of all hospitals in the region. The fourth step involves calculating the adjustment coefficient (AC). The AC includes both the base-level coefficient and the dynamic-level coefficient. The base level coefficient is set based on factors such as the hospital’s level, functional orientation, medical capabilities, and specialized features, reflecting the proportional relationship of average hospitalization expenditures across different hospitals for treating the same disease. The dynamic level coefficient is related to factors such as the severity of cases and patient age. The actual reimbursement for the hospital is the product of the point, PV, and AC.

## Methods

### The PBPS reform in public hospitals in City A

City A is located in the central region of Shandong Province, eastern China. As of 2023, the city had a population of 5.35 million. Its GDP reached 332.39 billion RMB, ranking 12th out of 16 cities in Shandong Province. By the end of 2023, City A was served by 112 hospitals, comprising 50 public and 62 private institutions. The number of hospital beds per 1,000 population in City A was 7.16, and the number of licensed physicians per 1,000 population was 3.30, both below the national average, reflecting the relatively underdeveloped economic status and healthcare infrastructure of City A compared to cities with high administrative rank and influence in China [39]. The medical insurance system in City A is primarily supported by two schemes: the Urban Employee Basic Medical Insurance (UEBMI) and the Urban Resident Basic Medical Insurance (URBMI), jointly covering over 96% of the city’s residents. A total of 5.147 million individuals were enrolled in the medical insurance system, with 1.219 million under UEBMI and 3.928 million under URBMI. Participants in the UEBMI scheme are typically employed individuals with higher income levels and better economic resources, granting them enhanced access to healthcare services compared to those insured under URBMI [40].

In November 2020, the National Healthcare Security Administration announced the first batch of pilot cities for implementing the DIP payment system, which included 71 cities across 27 provinces, with City A among them. These cities were required to complete the payment reform within 1–2 years. In January 2022, City A officially launched the DIP payment system, applying it to all inpatient cases in municipal hospitals (excluding psychiatric cases) to replace the previous FFS payment system.

Hospital Z is the largest public tertiary hospital in City A, responsible for treating more than half of the severe cases in the region. Since City A was designated as a pilot city for the DIP payment system, the city’s Healthcare Security Administration has regularly organized training sessions, inviting representatives from all local medical institutions to attend. These sessions aim to provide an interpretation of the DIP payment system and emphasize the urgency of implementing the PBPS reform in hospitals to promote more refined management under the DIP payment system.

In response to the requirements outlined by the Healthcare Security Administration, Hospital Z implemented an upgraded PBPS commencing in 2023. As delineated in Table 1, the revised PBPS introduces a new evaluation metric focusing on the efficiency of obtaining points (EOP) under the DIP payment system, which accounts for 15% of the total weighting. This modification represents a substantive enhancement compared to the pre-reform PBPS.

**Table 1:** Differences between the revised and original performance-based pay scheme (PBPS) at Hospital Z after 2023

Dimensions	Explanation
New assessment criteria	Efficiency of obtaining points (EOP)
Weight	15%
Scope of Application	All departments within the hospital, except for administrative departments.
Calculation method	The calculation method for EOP is the points of discharged cases divided by the medical expenses incurred. Using the hospital-wide average EOP as a benchmark, departments with an EOP above the average are assigned a score of 10%, while those with an EOP below the average are assigned a score of 5%. Additionally, the median monthly EOP of the assessed department over the previous six months serves as the benchmark. Departments experiencing a decline in EOP receive a score of 0, while for every 1% increase in EOP, the score increases by 1%. If the increase exceeds 5%, a maximum score of 5% is assigned.

### Data

The data for this study were sourced from the information management system of the City A Healthcare Security Administration. We utilized a comprehensive dataset that includes inpatient claims data from five tertiary public hospitals in City A, including Hospital Z, spanning the period from January 2022 to August 2024. This dataset contains beneficiaries from both the UEBMI and URBMI programs. Hospital Z implemented the PBPS reform in January 2023, with the period from January 2022 to December 2022 considered the pre-reform and the period from January 2023 to August 2024 considered the post-reform.

Although the Healthcare Security Administration of City A emphasizes the necessity of implementing the PBPS reform in hospitals, the other four tertiary public hospitals did not adopt the PBPS reform. This status was verified by the monitoring records and field evaluations conducted by the municipal government of City A, which indicated that these control hospitals continued to rely on comparable traditional revenue-based models due to insufficient understanding of the policy and inadequate management capacity [41]. Accordingly, 322,287 inpatient cases from Hospital Z were included in the intervention group, while 336,007 inpatient cases from the other four tertiary public hospitals were included in the control group. Cases with incomplete information on key variables were excluded from the analysis (*n* = 1872, 0.28% of total cases). The proportion of excluded cases was minimal and unlikely to materially affect the results.

The dataset includes basic demographic information of patients (such as age and gender), the name of the hospital, primary diagnoses based on ICD-10 codes, procedures based on ICD-9-CM3 codes, inpatient expenses, LOS, and other relevant information.

### Outcome variables

The primary outcome variables are hospitals’ revenue structure and service efficiency. Regarding the revenue structure, we examined the proportion of drug sales (PDS) and the proportion of laboratory tests and examinations (PLTE) in the total inpatient expenses. Regarding service efficiency, we observed the cost consumption index (CCI) and the time consumption index (TCI). The CCI reflects the treatment cost efficiency for similar diseases, while the TCI evaluates the time efficiency in treating similar diseases. The calculation formulas for these variables are as follows:

1 $$\:CCI=\frac{\sum\limits_{i=1}^{t}\left(\frac{\begin{aligned}& Average\:{cos}t\:for\:DIP\cr & grou{p}_{i}\:at\:the\:hospital\end{aligned}}{\begin{aligned}&Average\:{cos}t\:for\:DIP\:grou{p}_{i}\cr &across\:all\:hospitals\:in\:the\:region\end{aligned}}\text{}\times\:{N}_{i}\right)}{\begin{aligned}&The\:total\:Number\:of\cr & patients\:in\:the\:hospital\end{aligned}}$$

2 $$\:TCI=\frac{\sum\limits_{i=1}^{t}\left(\frac{\begin{aligned}& Average\:LOS\:for\:DIP\cr & grou{p}_{i}\:at\:the\:hospital\end{aligned}}{\begin{aligned}&Average\:LOS\:for\:DIP\:grou{p}_{i}\cr & across\:all\:hospitals\:in\:the\:region\end{aligned}}\text{}\times\:{N}_{i}\right)}{\begin{aligned}&The\:total\:Number\cr & of\:patients\:in\:the\:hospital\end{aligned}}$$

where *i* represents a specific DIP group, *t* refers to the number of DIP groups covered by the hospital’s services, and *N*_*i*_ represents number of cases in the DIP group *i* at the hospital. When the values of CCI and TCI are equal to 1, the hospital is comparable to the regional average level of medical institutions. A value less than 1 indicates lower medical costs or shorter LOS, while a value greater than 1 suggests higher medical costs or longer LOS. Compared to the per-case expense and LOS used in previous studies, these two variables enable cross-hospital comparisons, assessing hospitals’ service efficiency at a regional level and providing a comprehensive evaluation of overall operational management performance [42, 43].

### Statistical analysis

To reduce potential sources of bias, we employed a multiple-group controlled interrupted time series design to account for secular trends and baseline differences between hospitals, tested the parallel trends assumption prior to intervention, and conducted several sensitivity analyses to assess the robustness of the findings [44, 45]. The model was defined as follows:

3 $$\begin{aligned}{Y}_{t}=& \:{\beta}_{0}+{\beta}_{1}{T}_{t}+{\beta}_{2}{X}_{t}+{\beta}_{3}{X}_{t}{T}_{t}\cr & \quad \:+{\beta}_{4}I+{\beta}_{5}I{T}_{t}+{\beta}_{6}I{X}_{t}\cr & \quad \:+{\beta}_{7}I{X}_{t}{T}_{t}+{\epsilon}_{t}\end{aligned}$$

To facilitate interpretation, the specific meaning of each coefficient in the multiple-group CITS model is detailed in Table 2. Among these, $\:{\beta\:}_{6}$ and $\:{\beta\:}_{7}$ are the primary parameters of interest, representing the difference in the level change (immediate effect) and the difference in the slope change (long-term trend) between the intervention and control groups relative to the pre-intervention baseline.

**Table 2:** Interpretation of regression coefficients in the multiple-group CITS model

Coefficient	Term	Interpretation
**Control Group**		
$\:{\beta\:}_{0}$	Intercept	Baseline level of the outcome in the control group.
$\:{\beta\:}_{1}$	$\:{T}_{t}$	Baseline trend (slope) of the outcome in the control group.
$\:{\beta\:}_{2}$	$\:{X}_{t}$	Level change in the control group immediately after the intervention time point.
$\:{\beta\:}_{3}$	$\:{X}_{t}{T}_{t}$	Trend change (slope change) in the control group after the intervention time point.
**Intervention Group**		
$\:{\beta\:}_{4}$	$\:I$	Difference in the baseline level between the intervention and control groups.
$\:{\beta\:}_{5}$	$\:I{T}_{t}$	Difference in the baseline trend between the intervention and control groups (Test for Parallel Trends).
$\:{\beta\:}_{6}$	$\:I{X}_{t}$	Difference in the level change between the intervention and control groups immediately after the reform (Immediate Policy Effect).
$\:{\beta\:}_{7}$	$\:I{X}_{t}{T}_{t}$	Difference in the trend change between the intervention and control groups after the reform (Long-term Policy Effect).
$\:{\epsilon\:}_{t}$		error term

The validity of the control group was statistically confirmed by testing the parallel trends assumption; as shown in Table S1, the pre-intervention slope difference ($\:{\beta\:}_{5}$) between the intervention and control groups was not statistically significant for any outcome measure, satisfying the methodological requirement for comparability [46]. Furthermore, a continuous time variable ($\:{T}_{t}$) was utilized instead of year-specific indicators to capture continuous trend changes and to avoid perfect multicollinearity with the intervention variable ($\:{X}_{t}$), given that the reform coincided with the start of the calendar year [47].

We applied a Prais-Winsten estimation with the Durbin-Watson statistic to correct autocorrelation and employed robust standard errors. We first examined all cases covered by medical insurance. Subsequently, we conducted a stratified analysis for patients covered under the UEBMI and URBMI programs to determine whether the effect of the PBPS reform varied by insurance type. All statistical analyses were performed using Stata/MP 17.0.

## Results

### Sample characteristics

Our study included 658,294 inpatient cases from five hospitals in City A between January 2022 and August 2024. Table 3 presents the sample characteristics of patients in the intervention and control groups before and after the PBPS reform. The average age of patients in both groups is around 55 years (*P* < 0.001), with a slightly higher proportion of male patients compared to female patients. Over 60% of the patients are enrolled in the URBMI program, and the composition of insurance types differed significantly between the two groups (*P* < 0.001). After the PBPS reform, the PDS decreased, and the PLTE increased in both the intervention and control groups. Meanwhile, the CCI and TCI decreased in both groups.

**Table 3:** Descriptive statistics of patients in the intervention and control groups before and after the performance-based pay schemes reform

Category	Before PBPS reform		After PBPS reform		
	Intervention	Control	Intervention	Control	
**Patient characteristics**					P-value
Sample size	95,031	104,496	227,256	231,511	
Age, mean (SD)	55.13(20.43)	54.26(20.05)	56.22(20.82)	55.71(20.65)	< 0.001
Male sex, No. (%)	48,021(50.53)	53,356(51.06)	115,283(50.73)	119,020(51.41)	< 0.001
Insurance type					
UEBMI, No. (%)	36,333(38.23)	39,019(37.34)	87,129(38.34)	90,592(39.13)	< 0.001
URBMI, No. (%)	58,698(61.77)	65,477(62.66)	140,127(61.66)	140,919(60.87)	
**Patient outcomes**					
Revenue structure					
PDS (%)	28.38	25.72	24.32	23.48	
PLTE (%)	25.38	29.53	28.01	31.52	
Service efficiency					
CCI	1.15	1.11	0.93	0.88	
TCI	0.86	0.99	0.78	0.86	

Note: PBPS, Performance-based pay schemes; SD, Standard deviation; UEBMI, Urban Employee Basic Medical Insurance; URBMI, Urban Resident Basic Medical Insurance; PDS, Proportion of drug sales in total inpatient expenses; PLTE, Proportion of laboratory tests and examinations in total inpatient expenses; CCI, Cost consumption index; TCI, Time consumption index

### Revenue structure

#### Proportion of drug sales in total inpatient expenses

**Fig. 2:**
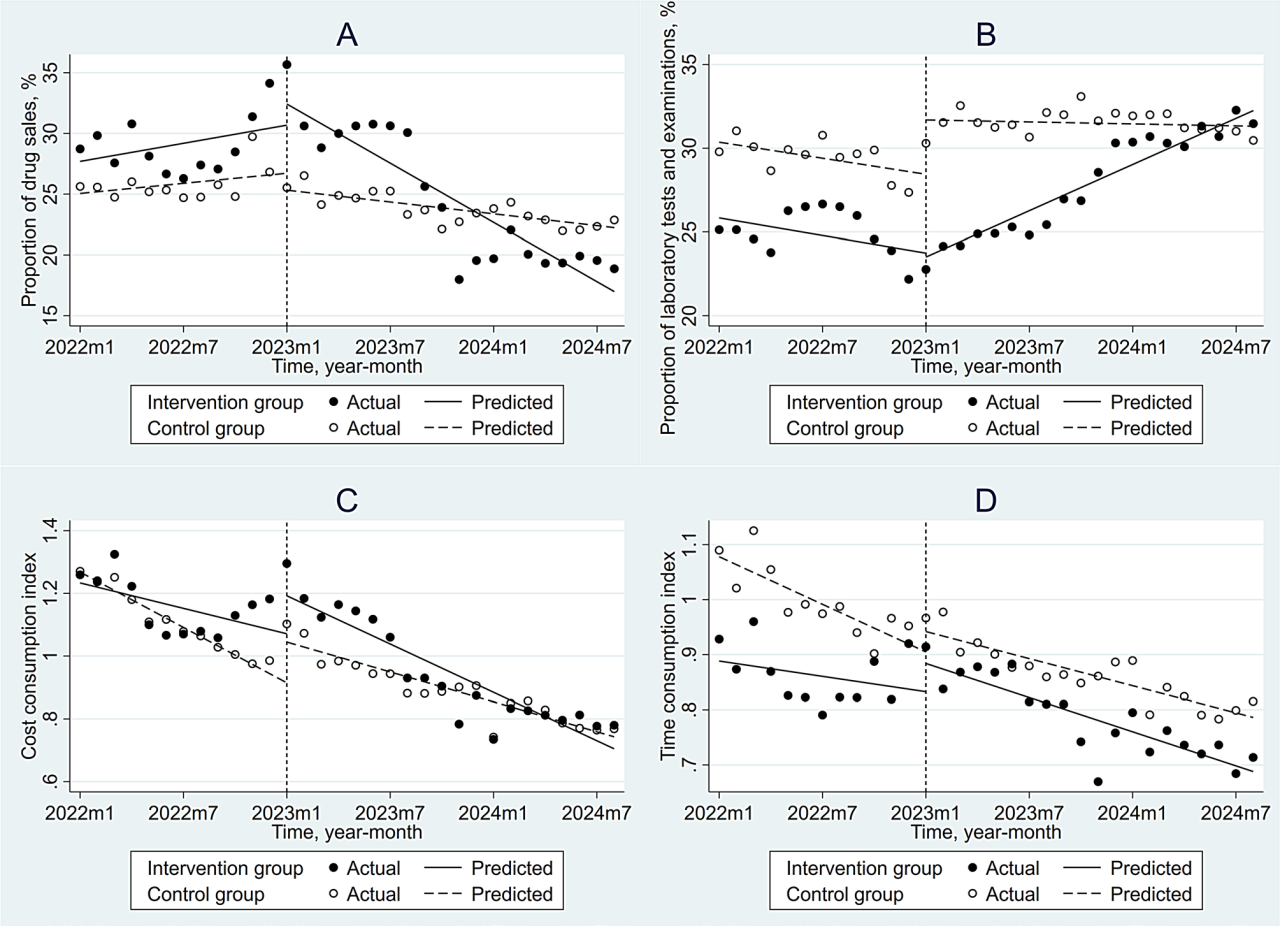
Controlled interrupted time series plots for public hospitals in City A. (**A**) is the proportion of drug sales in total inpatient expenses; (**B**) is the proportion of laboratory tests and examinations in total inpatient expenses; (**C**) is the cost consumption index; (**D**) is the time consumption index

Table 4 presents the immediate and trend effects of the PBPS reform on the full sample and subgroups stratified by insurance type based on the CITS analysis results. Before the PBPS reform, there were no statistically significant differences in the level or trend of PDS between the intervention and control groups (Table S1). After the PBPS reform, there was no immediate change in PDS in the intervention group compared to the control group, either in the overall sample or within the subgroups. However, compared to the control group, the monthly trend of PDS in the intervention group significantly decreased by 0.76% in the overall sample (*P* = 0.046) and by 0.875% in the subgroup of patients covered by UEBMI (*P* = 0.035), while no significant difference was observed in the subgroup of patients covered by URBMI. Figure 2 A, Figure S1A, and Figure S2A illustrate the effects of the PBPS reform on PDS in the overall sample and subgroups stratified by insurance type, respectively.

**Table 4:** Controlled interrupted time series analyses for performance-based pay schemes reform in City A

Insurance type	Variables	Difference in Step Change Between Groups		Difference in Trend Change Between Groups	
		*β*_*6*_	(95%CI)	*β*_*7*_	(95%CI)
Overall	PDS	3.140	(-1.657, 7.936)	-0.760*	(-1.508, -0.012)
	PLTE	-3.479**	(-5.922, -1.036)	0.495**	(0.127, 0.863)
	CCI	-0.009	(-0.156, 0.139)	-0.026*	(-0.048, -0.003)
	TCI	0.014	(-0.073, 0.101)	-0.012*	(-0.023, -0.001)
UEBMI	PDS	3.677	(-1.631, 8.986)	-0.875*	(-1.687, -0.063)
	PLTE	-4.246**	(-7.208, -1.284)	0.503*	(0.104, 0.902)
	CCI	0.065	(-0.089, 0.219)	-0.026*	(-0.050, -0.001)
	TCI	0.017	(-0.093, 0.127)	-0.015*	(-0.029, -0.001)
URBMI	PDS	2.882	(-1.972, 7.736)	-0.691	(-1.412, 0.031)
	PLTE	-2.922*	(-5.462, -0.381)	0.461*	(0.090, 0.832)
	CCI	-0.049	(-0.206, 0.107)	-0.026*	(-0.049, -0.003)
	TCI	0.012	(-0.072, 0.096)	-0.011*	(-0.021, -0.001)

Note: UEBMI, Urban Employee Basic Medical Insurance; URBMI, Urban Resident Basic Medical Insurance; PDS, Proportion of drug sales in total inpatient expenses; PLTE, Proportion of laboratory tests and examinations in total inpatient expenses; CCI, Cost consumption index; TCI, Time consumption index. * *p* < 0.05; ** *p* < 0.01; *** *p* < 0.001

#### Proportion of laboratory tests and examinations in total inpatient expenses

As shown in Table S1, before the intervention, although there was a significant difference in the initial level of PLTE between the intervention and control groups (*β*_*4*_ = 3.248, *P* < 0.001), no significant difference in the trends was observed between the two groups. Following the PBPS reform, in the overall sample, the difference in the immediate level change between the two groups was − 3.479% (*P* = 0.006). Sensitivity analysis (Table S4) indicates that this was a protective effect: the reform prevented the immediate increase in PLTE that occurred in the control hospitals, resulting in a significant relative reduction. Following this immediate shift, the PLTE showed a relative upward trend (${\beta _7}$= 0.495, *P* = 0.009), contributing to the V-shaped trajectory observed over time. In the subgroups covered by UEBMI and URBMI, PLTE decreased immediately by 4.246% (*P* = 0.006) and 2.922% (*P* = 0.025) compared to the control group, respectively, while the monthly trends increased by 0.503% (*P* = 0.014) and 0.461% (*P* = 0.016) compared to the control group, respectively. Figure 2B, Figure S1B, and Figure S2B illustrate the effects of the PBPS reform on PLTE in the overall sample and subgroups stratified by insurance type, respectively.

### Service efficiency

#### Cost consumption index

As indicated in Table S1, before the implementation of the policy intervention, the intervention and control groups exhibited a significant difference in initial levels (*β*_*4*_ = 0.13, *P* = 0.015) of CCI; however, no statistically significant difference was detected in the pre-intervention trends between the two groups. Following the PBPS reform, there was no immediate change in CCI for the intervention group compared to the control group in the overall sample; however, the monthly trend showed a significant decline of 0.026 (*P* = 0.025) compared to the control group. Similarly, in the subgroups of patients covered by UEBMI and URBMI, no immediate change in CCI was observed post-reform, while the monthly trends significantly decreased by 0.026 (*P* = 0.040 and *P* = 0.027, respectively) compared to the control group in both subgroups. Figure 2 C, Figure S1C, and Figure S2C illustrate the effects of the PBPS reform on CCI in the overall sample and subgroups stratified by insurance type, respectively.

#### Time consumption index

As shown in Table S1, the differences in TCI levels and slopes between the intervention and control groups were insignificant before the policy intervention. After the PBPS reform, in the overall sample, there was no immediate change in TCI for the intervention group compared to the control group; however, the monthly trend showed a significant decline of 0.012 (*P* = 0.032) compared to the control group. Similarly, in the subgroups of patients covered by UEBMI and URBMI, no immediate changes in TCI were observed after the reform, while the monthly trends significantly decreased by 0.015 (*P* = 0.036) and 0.011 (*P* = 0.045) compared to the control group, respectively. Figure 2D, Figure S1D, and Figure S2D illustrate the effects of the PBPS reform on TCI in the overall sample and subgroups stratified by insurance type, respectively.

### Sensitivity analyses

We performed three sensitivity analyses. First, we excluded patients admitted in 2022 and discharged in 2023, as it was unclear whether the upgraded PBPS was used to calculate the incentives for treating these patients (Table S2). Second, we excluded patients who were transferred from other hospitals, as they may have received part of their treatment (e.g., surgery) at the original hospital, and the outcomes may not accurately reflect the hospital’s revenue structure and efficiency (Table S3). Third, to further validate the attribution of the observed effects to the PBPS reform and address potential heterogeneity, we conducted separate single-group ITS analyses for the intervention hospital and each of the four control hospitals individually. As presented in Table S4, Hospital Z consistently exhibited the most pronounced improvements following the intervention. In contrast, the four control hospitals generally showed either non-significant changes, trends in the opposite direction, or significantly smaller magnitudes of change compared to Hospital Z. This distinct divergence confirms that the observed improvements in revenue structure and efficiency were unique to the intervention hospital and not driven by general secular trends affecting the region.

## Discussion

This study provides empirical evidence on how aligning internal incentives through PBPS reform under the DIP payment system can affect the revenue structure and service efficiency of Chinese public hospitals, which was originally introduced as a coding-feasible case-based model intended to promote more cost-effective care in such settings.

Our findings indicate that following the PBPS reform, the monthly trend of PDS declined significantly by 0.76% relative to the control group. Sensitivity analysis (Table S4) further confirms that the intervention hospital exhibited the steepest downward trend in drug expenditure share (${\beta _3}$= -1.059, *P* < 0.001) compared to the control hospitals. However, we observed that this effect was heterogeneous and primarily driven by the subgroup of patients covered by UEBMI, suggesting that the optimization of revenue structure was most pronounced where insurance coverage and payment incentives were strongest, rather than being uniform across all patient populations. Under the previous FFS system, overprescription was economically incentivized, as drug revenues directly contributed to departmental bonuses [48]. The DIP payment system, by contrast, introduces fixed payments based on points, thereby limiting the marginal financial return from drug utilization. When PBPS is redesigned to reward efficiency, this alignment reduces incentives for unnecessary medication.

The reduction in drug expenditure is beneficial from multiple perspectives. Clinically, it encourages more rational prescribing practices and stricter adherence to treatment pathways, reducing the risks of polypharmacy, adverse drug events, and antimicrobial resistance—issues that pose increasingly serious public health challenges in China and globally [49]. Although cost considerations initially drive these changes, they may lead to long-term improvements in healthcare quality and patient safety. Economically, it reduces the financial burden on patients, particularly those with chronic diseases or those requiring long-term hospitalization [50].

However, the impact differed by insurance type. The decline in PDS was more pronounced among UEBMI beneficiaries than among those covered by URBMI. These insurance-related differences primarily reflect long-standing demographic and socioeconomic distinctions between UEBMI and URBMI beneficiaries, rather than changes in service volume induced by the DIP payment mechanism. UEBMI patients typically benefit from more comprehensive coverage and may be treated in hospitals that offer greater flexibility in adjusting prescribing behavior. They are also more likely to be working-age individuals with higher health literacy, making them more receptive to optimized treatment regimens [51]. In contrast, URBMI often covers older adults and low-income populations who may have more complex conditions and higher medication needs, limiting the potential for short-term reductions in drug use [52]. These findings suggest that, while the reform improves the revenue structure of public hospitals, further adjustments may be required to prevent unintended equity gaps across insurance groups.

We also found that the PLTE exhibited a significant relative immediate reduction at the onset of the PBPS reform, followed by a relative monthly upward trend. As clarified by our sensitivity analysis, this immediate reduction was driven by the intervention preventing the cost surge observed in the control group. However, the subsequent upward trend suggests a potential strategic shift in hospitals’ behavior to compensate for the new constraints. Under the DIP payment system, hospitals are incentivized to increase their service volume to secure a larger share of the fixed global budget, which is allocated based on points [20]. This incentive structure has inadvertently led to unnecessary hospital admissions, including those for low-threshold and health check-ups [53]. In our study, the similarity with findings from Guangzhou and Beijing lies in the presence of such mismatches, rather than the specific downstream behavioral responses observed in those regions. Our inpatient dataset does not capture outpatient substitution or cost-shifting, and therefore we do not infer that such adjustments have occurred in the present setting. However, we can offer a plausible explanation for this unexpected outcome. Physicians in hospitals implementing PBPS reform are subject to stronger economic incentives, further intensifying their motivation to generate more claimable points. To increase the number of inpatient cases and associated point-based reimbursements, some hospitals may admit patients with mild conditions who could have been managed in outpatient settings or convert health check-ups into inpatient admissions. These practices artificially inflate inpatient volumes while minimizing therapeutic intensity and per-case cost, which may explain the observed upward trend in PLTE following the PBPS reform. Our results further show that, compared to hospitals in the control group, those in the intervention group experienced a significant monthly increase in inpatient volumes after the PBPS reform (Table S5), which indirectly supports the possibility of such behavior. While such strategies might improve a hospital’s short-term financial performance under the DIP payment system, they raise concerns about the rationality of resource allocation [54]. These patterns likely reflect multiple interacting factors during the early implementation of DIP, rather than a single dominant mechanism. Importantly, the increase in PLTE was observed in both insurance groups, suggesting a system-wide shift in service composition during the early stages of DIP implementation rather than an insurance-specific increase in utilization. These findings underscore the need for complementary regulatory mechanisms to monitor admission appropriateness, enhance clinical auditing, and prevent systemic gaming, thereby ensuring that the PBPS reform leads to improvements in service quality rather than superficial adjustments in the service mix.

Both the CCI and TCI exhibited significant downward trends following the PBPS reform, suggesting improved efficiency. By rewarding efficiency, hospitals were incentivized to deliver necessary services more streamlined, potentially improving the utilization of medical resources and reducing unnecessary inpatient days or redundant procedures [55]. However, such changes should also be interpreted cautiously. Declining CCI and TCI may also signal a strategic response to minimize the cost and time inputs per case, which could result in potential under-treatment, particularly in more complex or chronic cases [56]. Therefore, future reforms should consider integrating quality-monitoring mechanisms into the design of PBPS to ensure that cost and time efficiency improvements do not compromise patient safety.

Taken together, these significant shifts across multiple indicators suggest that hospitals actively adjusted their service delivery in response to the DIP-aligned PBPS reform, consistent with the intended role of payment incentives in shaping hospitals’ behavior.

This study has several limitations. First, as previously discussed, improving healthcare quality is a critical criterion for evaluating the success of medical reform. However, we were unable to obtain direct healthcare quality measures due to data availability limitations. Second, under the DIP payment system, the regulatory mechanism of the Healthcare Security Administration is still under development, and there is no standardized definition or regulation for practices such as low-threshold admissions or health check-up admissions. As a result, we could not directly quantify the extent to which such potentially unnecessary admissions were attributable to the reform. In addition, the studies referenced in the Discussion to illustrate possible explanations for these patterns were cited solely to provide contextual interpretation rather than to draw inferential conclusions, and should not be viewed as evidence that such practices occurred in our setting. Third, caution should be exercised when generalizing our findings to the national level. Significant regional disparities in healthcare capacity and the allocation of medical resources across China may lead to heterogeneous effects of the reform in different local contexts. City A represents a mid-level prefecture city rather than a top-tier metropolitan area, and its institutional capacity, degree of hospital competition, and policy implementation intensity may differ from those in larger or more developed regions. Therefore, the magnitude of the observed effects may not be directly transferable to all settings. However, it is worth noting that the DIP payment system was intentionally designed as a scalable model suitable for regions with varying levels of technical readiness and administrative capacity. In this sense, our findings may be particularly relevant for similar mid-level cities undergoing internal incentive alignment under case-based payment reform. Future multi-city or national-level studies are warranted to further assess external validity.

## Conclusion

This study provides empirical evidence on the impact of PBPS reform under the DIP payment system in China. The reform was associated with a significant decline in PDS and improvements in service efficiency, as indicated by reduced CCI and TCI. However, the differential effects observed between UEBMI and URBMI beneficiaries raise essential concerns regarding health equity. Moreover, the observed increase in PLTE and service volume suggests potential unintended consequences. These findings underscore the importance of aligning internal incentive mechanisms with the case-based payment system to ensure cost-effective healthcare delivery.

## Supplementary Information

Below is the link to the electronic supplementary material.

Supplementary Material 1

## Data Availability

The data analyzed in this study is subject to the following licenses/restrictions: the data that support the findings of this study are available from the Healthcare Security Administrations, but restrictions apply to the availability of these data, which were used under license for the current study, and so are not publicly available. Requests to access these datasets should be directed to HS, (mailto: hyshi@buaa.edu.cn).

## Abbreviations

FFS: Fee-for-Service
DIP: Diagnosis-Intervention Package
DRGs: Diagnosis-Related Groups
PBPS: Performance-based pay schemes
LOS: Lengths of stay
PV: Point value
AC: Adjustment coefficient
EOP: Efficiency of obtaining points
UEBMI: Urban Employee Basic Medical Insurance
URBMI: Urban Resident Basic Medical Insurance
PDS: Proportion of drug sales in total inpatient expenses
PLTE: Proportion of laboratory tests and examinations in total inpatient expenses
CCI: Cost consumption index
TCI: Time consumption index
