# Supplementary material for: The impact of public hospital performance-based pay scheme reform on revenue structure and service efficiency under the Diagnosis-Intervention Packet payment system: evidence from a pilot city in China
Source: BMC Health Serv Res. 2026 Mar 28;26:650. doi: 10.1186/s12913-026-14449-7 (PMC13151393; doi:10.1186/s12913-026-14449-7)
Supplement: Supplementary file 1 — Supplementary Material 1 [file 12913_2026_14449_MOESM1_ESM.docx]

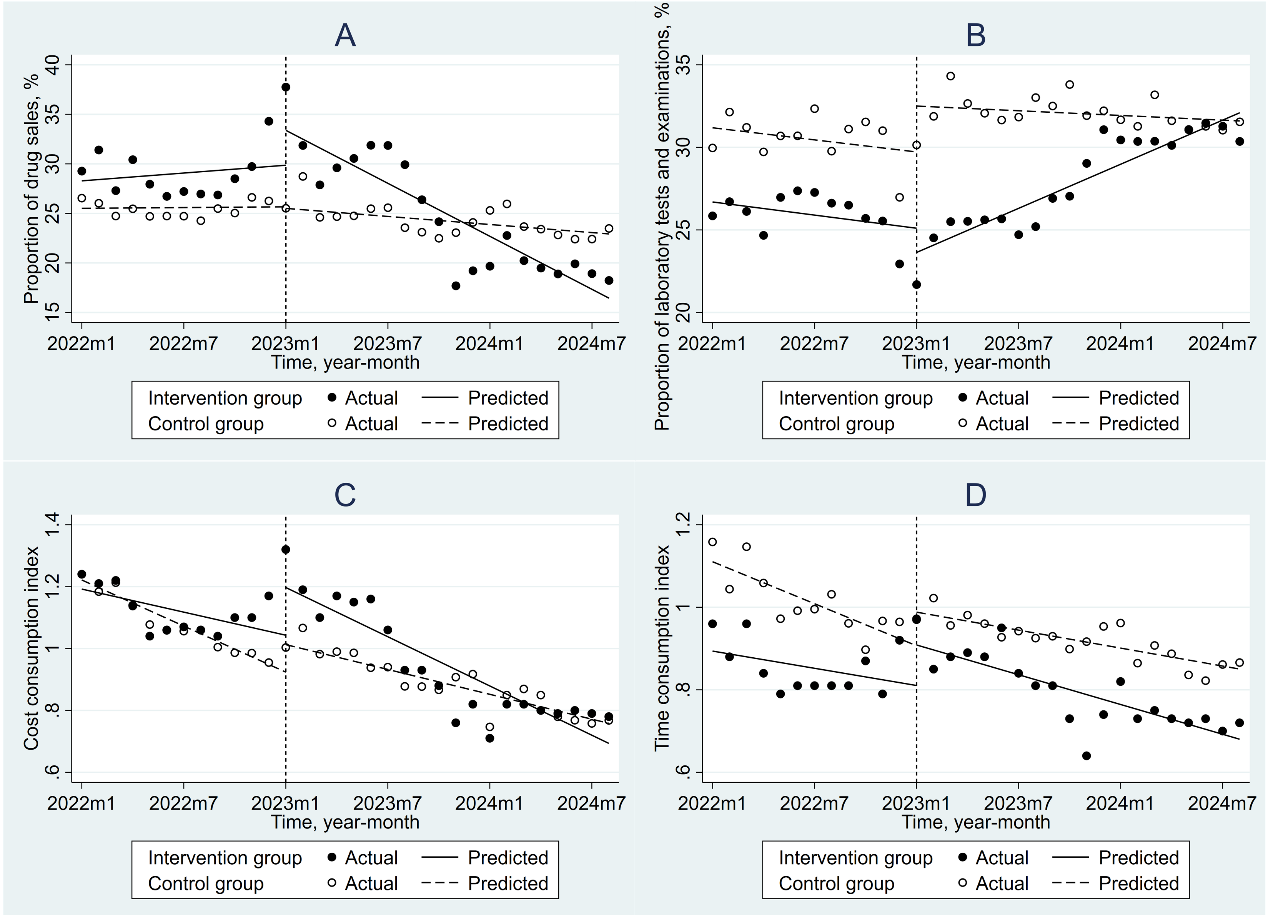


**Figure S1** Controlled interrupted time series plots for the subgroup of UEBMI-covered patients in public hospitals in City A. **(A)** is the proportion of drug sales in total inpatient expenses; **(B)** is the proportion of laboratory tests and examinations in total inpatient expenses; **(C)** is the cost consumption index; **(D)** is the time consumption index.


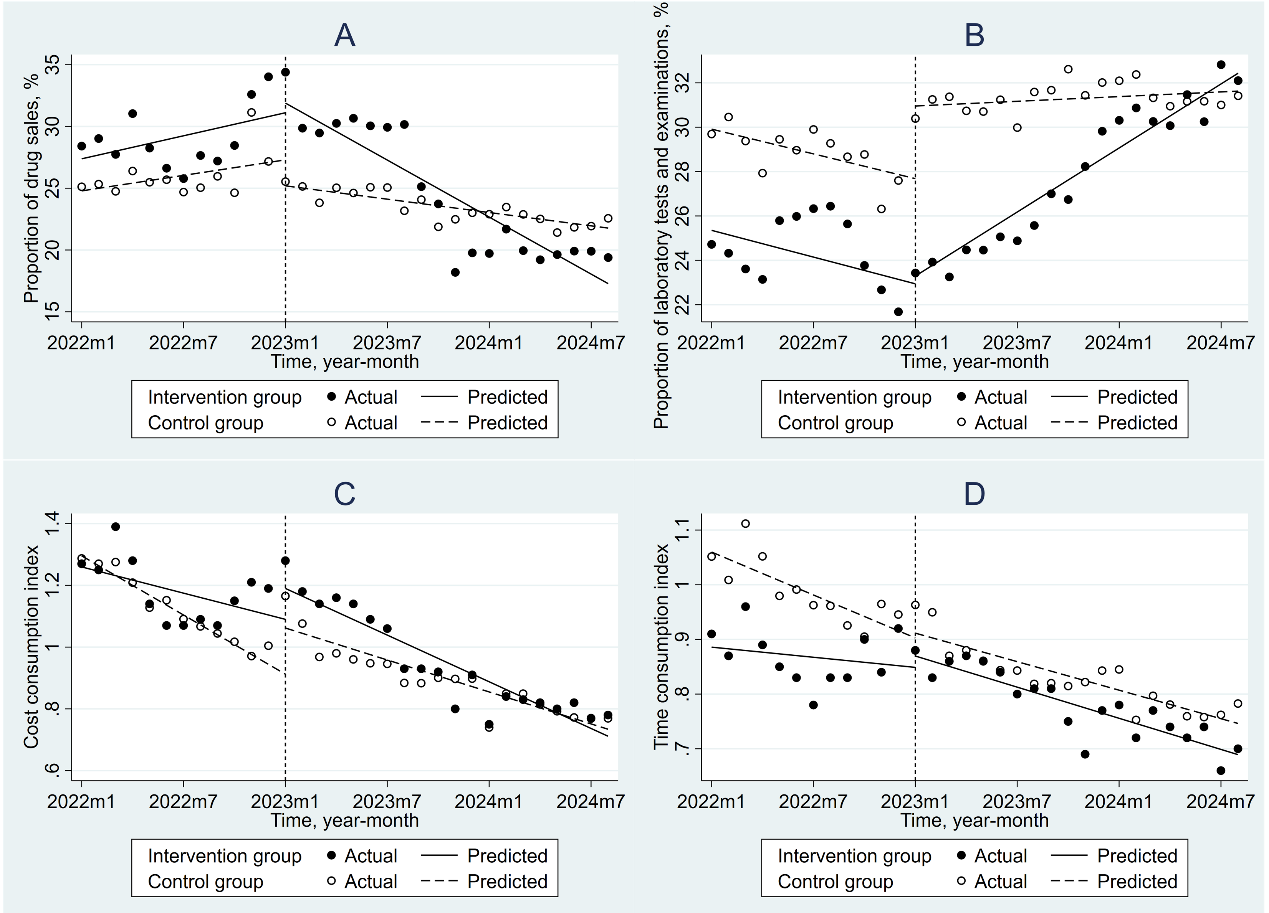


**Figure S2** Controlled interrupted time series plots for the subgroup of URBMI-covered patients in public hospitals in City A. **(A)** is the proportion of drug sales in total inpatient expenses; **(B)** is the proportion of laboratory tests and examinations in total inpatient expenses; **(C)** is the cost consumption index; **(D)** is the time consumption index.

**Table S1** Controlled interrupted time series estimates other than *β_6_* and *β_7_*

|  | *β_0_* | *β_1_* | *β_2_* | *β_3_* | *β_4_* | *β_5_* |
| --- | --- | --- | --- | --- | --- | --- |
| **Overall** |  |  |  |  |  |  |
| PDS | 25.053*** | 0.139 | 2.640 | 0.110 | -1.397 | -0.301 |
| PLTE | 30.360*** | -0.160 | -4.526*** | -0.015 | 3.248*** | 0.140 |
| CCI | 1.268*** | -0.029*** | -0.035 | 0.016 | 0.130* | 0.014 |
| TCI | 1.078*** | -0.014*** | -0.190*** | 0.010* | 0.037 | 0.006 |
| **UEBMI** |  |  |  |  |  |  |
| PDS | 25.514*** | 0.013 | 2.772 | 0.119 | -0.156 | -0.148 |
| PLTE | 31.187*** | -0.122 | -4.494*** | -0.010 | 2.781** | 0.075 |
| CCI | 1.222*** | -0.025** | -0.029 | 0.012 | 0.089 | 0.011 |
| TCI | 1.110*** | -0.017*** | -0.217*** | 0.010 | 0.081* | 0.010 |
| **URBMI** |  |  |  |  |  |  |
| PDS | 24.785*** | 0.209 | 2.594 | 0.102 | -2.103 | -0.389 |
| PLTE | 29.914*** | -0.186 | -4.563*** | -0.015 | 3.275** | 0.221 |
| CCI | 1.296*** | -0.032*** | -0.036 | 0.018 | 0.150** | 0.015 |
| TCI | 1.060*** | -0.013*** | -0.174*** | 0.010* | 0.009 | 0.004 |

Note: UEBMI, Urban Employee Basic Medical Insurance; URBMI, Urban Resident Basic Medical Insurance; PDS, Proportion of drug sales in total inpatient expenses; PLTE, Proportion of laboratory tests and examinations in total inpatient expenses; CCI, Cost consumption index; TCI, Time consumption index.

* p<0.05; ** p<0.01; *** p<0.001

**Table S2** Sensitivity analysis: excluding cases admitted in 2022 and discharged in 2023

| Insurance type | Variables | Difference in Step Change Between Groups | | Difference in Trend Change Between Groups | |
| --- | --- | --- | --- | --- | --- |
|  |  | *β_6_* | (95%CI) | *β_7_* | (95%CI) |
| Overall | PDS | 3.336 | (-1.469, 8.141) | -0.760* | (-1.507, -0.012) |
|  | PLTE | -3.608** | (-6.042, -1.173) | 0.495** | (0.129, 0.860) |
|  | CCI | 0.001 | (-0.146, 0.148) | -0.026* | (-0.048, -0.003) |
|  | TCI | 0.019 | (-0.068, 0.106) | -0.012* | (-0.023, -0.001) |
| UEBMI | PDS | 3.860 | (-1.464, 9.185) | -0.874* | (-1.686, -0.062) |
|  | PLTE | -4.325** | (-7.283, -1.367) | 0.504* | (0.107, 0.902) |
|  | CCI | 0.069 | (-0.085, 0.224) | -0.026* | (-0.050, -0.001) |
|  | TCI | 0.022 | (-0.089, 0.132) | -0.015* | (-0.029, -0.001) |
| URBMI | PDS | 3.047 | (-1.807, 7.902) | -0.691 | (-1.411, 0.031) |
|  | PLTE | -3.044* | (-5.574, -0.514) | 0.460* | (0.091, 0.829) |
|  | CCI | -0.036 | (-0.191, 0.118) | -0.026* | (-0.048, -0.003) |
|  | TCI | 0.018 | (-0.065, 0.101) | -0.011* | (-0.021, -0.001) |

Note: UEBMI, Urban Employee Basic Medical Insurance; URBMI, Urban Resident Basic Medical Insurance; PDS, Proportion of drug sales in total inpatient expenses; PLTE, Proportion of laboratory tests and examinations in total inpatient expenses; CCI, Cost consumption index; TCI, Time consumption index.

* p<0.05; ** p<0.01; *** p<0.001

**Table S3** Sensitivity analysis: excluding cases transferred from other hospitals

| Insurance type | Variables | Difference in Step Change Between Groups | | Difference in Trend Change Between Groups | |
| --- | --- | --- | --- | --- | --- |
|  |  | *β_6_* | (95%CI) | *β_7_* | (95%CI) |
| Overall | PDS | 3.227 | (-1.564, 8.018) | -0.765* | (-1.512, -0.018) |
|  | PLTE | -3.466** | (-5.912, -1.020) | 0.494* | (0.125, 0.863) |
|  | CCI | -0.010 | (-0.157, 0.138) | -0.026* | (-0.049, -0.003) |
|  | TCI | 0.019 | (-0.065, 0.104) | -0.012* | (-0.022, -0.001) |
| UEBMI | PDS | 3.757 | (-1.555, 9.069) | -0.878* | (-1.690, -0.066) |
|  | PLTE | -4.242** | (-7.205, -1.278) | 0.502* | (0.103, 0.901) |
|  | CCI | 0.064 | (-0.090, 0.217) | -0.026* | (-0.050, -0.002) |
|  | TCI | 0.021 | (-0.088, 0.129) | -0.015* | (-0.028, -0.001) |
| URBMI | PDS | 2.962 | (-1.885, 7.809) | -0.694 | (-1.414, 0.026) |
|  | PLTE | -2.896* | (-5.443, -0.350) | 0.460* | (0.087, 0.834) |
|  | CCI | -0.050 | (-0.207, 0.107) | -0.026* | (-0.049, -0.003) |
|  | TCI | 0.020 | (-0.063, 0.103) | -0.011* | (-0.021, -0.001) |

Note: UEBMI, Urban Employee Basic Medical Insurance; URBMI, Urban Resident Basic Medical Insurance; PDS, Proportion of drug sales in total inpatient expenses; PLTE, Proportion of laboratory tests and examinations in total inpatient expenses; CCI, Cost consumption index; TCI, Time consumption index.

* p<0.05; ** p<0.01; *** p<0.001

**Table S4** Sensitivity analysis: Single-group ITS estimates for the intervention hospital (Z) and control hospitals (A-D)

| Hospital | Variables | Baseline slope β_1_ (95%CI) | Step change β_2_ (95%CI) | Slope change β_3_ (95%CI) |
| --- | --- | --- | --- | --- |
| Z | PDS | 0.205 (-0.220,0.630) | 2.565 (-1.553, 6.682) | -1.059 (-1.516, -0.603)*** |
|  | PLTE | -0.111 (-0.343,0.121) | -1.302 (-3.330, 0.726) | 0.611 (0.373, 0.849)*** |
|  | CCI | -0.013 (-0.025,-0.001)* | 0.124 (0.012, 0.235)* | -0.013 (-0.027, 0.001)* |
|  | TCI | -0.002 (-0.010,0.007) | 0.023 (-0.040, 0.087) | -0.009 (-0.017, -0.001)* |
| A | PDS | 0.074 (-0.066,0.213) | -0.671 (-3.516, 2.174) | -0.276 (-0.521, -0.032)* |
|  | PLTE | 0.042 (-0.119,0.203) | 2.490 (0.350, 4.631)* | -0.038 (-0.246, 0.170) |
|  | CCI | -0.016 (-0.019,-0.012)*** | 0.094 (0.041, 0.146)** | 0.001 (-0.004, 0.006) |
|  | TCI | -0.009 (-0.013,-0.005)*** | 0.028 (-0.020, 0.077) | 0.002 (-0.003, 0.008) |
| B | PDS | 0.295 (-0.115,0.705) | 1.897 (-2.470, 6.265) | -0.878 (-1.346, -0.409)** |
|  | PLTE | -0.214 (-0.374,-0.054)* | 1.158 (-0.269, 2.585) | 0.405 (0.214, 0.596)*** |
|  | CCI | -0.036 (-0.044,-0.029)*** | 0.061 (-0.012, 0.134) | 0.013 (0.004, 0.021)** |
|  | TCI | -0.027 (-0.037,-0.016)*** | 0.128 (0.056, 0.199)** | 0.012 (0.002, 0.023)* |
| C | PDS | -0.660 (-1.494,0.175) | 4.560 (-0.068, 9.188) | 0.412 (-0.454, 1.277) |
|  | PLTE | -0.021 (-0.286,0.245) | 0.069 (-2.352, 2.491) | -0.052 (-0.360, 0.255) |
|  | CCI | -0.059 (-0.068,-0.049)*** | 0.112 (0.020, 0.203)* | 0.054 (0.044, 0.063)*** |
|  | TCI | -0.054 (-0.064,-0.044)*** | 0.044 (-0.043, 0.131) | 0.053 (0.043, 0.064)*** |
| D | PDS | 0.491 (-0.346,1.328) | -3.112 (-11.183, 4.960) | -0.745 (-1.621, 0.131) |
|  | PLTE | -0.906 (-1.474,-0.337)** | 10.088 (4.451, 15.725)** | 0.464 (-0.164, 1.093) |
|  | CCI | -0.034 (-0.053,-0.014)** | 0.240 (0.113, 0.367)** | 0.028 (0.007, 0.049)* |
|  | TCI | -0.010 (-0.019,-0.001)* | 0.154 (0.062, 0.246)** | 0.014 (0.003, 0.024)* |

Note: PDS, Proportion of drug sales in total inpatient expenses; PLTE, Proportion of laboratory tests and examinations in total inpatient expenses; CCI, Cost consumption index; TCI, Time consumption index.

Hospital Z is the intervention hospital, and hospitals A, B, C, and D are four control hospitals.

The standard single-group ITS regression model assumes the following form: $Y_{t}=\beta_{0}+\beta_{1}T_{t}+\beta_{2}X_{t}+\beta_{3}X_{t}T_{t}+\varepsilon_{t}$, where $Y_{t}$ is the outcome variable measured at each time interval *t*, $T_{t}$ is the time since the beginning of the study; $X_{t}$ is a dummy variable representing the intervention, which is 0 before intervention and 1 after intervention; $X_{t}T_{t}$ is an interaction term; $\beta_{0}$ represents the baseline level of the outcome variable; $\beta_{1}$ is the slope of the outcome variable before the intervention; $\beta_{2}$ is the level change of the outcome variable after the intervention, and $\beta_{3}$ represents the slope change of the outcome variable after the intervention.

* p<0.05; ** p<0.01; *** p<0.001

**Table S5** Controlled interrupted time series analysis of inpatient volumes in hospitals in City A

| Insurance type | Difference in Step Change Between Groups | | Difference in Trend Change Between Groups | |
| --- | --- | --- | --- | --- |
|  | *β_6_* | (95%CI) | *β_7_* | (95%CI) |
| Overall | 1555.033 | (-1407.965, 4518.031) | 462.875* | (100.830, 824.921) |
| UEBMI | 279.387 | (-963.227, 1522.002) | 159.775* | (8.480, 311.069) |
| URBMI | 1132.634 | (-934.182, 3199.450) | 293.680* | (36.584, 550.776) |

Note: UEBMI, Urban Employee Basic Medical Insurance; URBMI, Urban Resident Basic Medical Insurance.

* p<0.05; ** p<0.01; *** p<0.001

**Table S6** STROBE checklist

|  | Item No. | Recommendation | Page  No. |
| --- | --- | --- | --- |
| **Title and abstract** | 1 | (*a*) Indicate the study’s design with a commonly used term in the title or the abstract | 1 |
|  |  | (*b*) Provide in the abstract an informative and balanced summary of what was done and what was found | 1-2 |
| Introduction | | | |
| Background/rationale | 2 | Explain the scientific background and rationale for the investigation being reported | 4-5 |
| Objectives | 3 | State specific objectives, including any prespecified hypotheses | 5-6 |
| Methods | | | |
| Study design | 4 | Present key elements of study design early in the paper | 5,11 |
| Setting | 5 | Describe the setting, locations, and relevant dates, including periods of recruitment, exposure, follow-up, and data collection | 7-10 |
| Participants | 6 | (*a*) *Cohort study*—Give the eligibility criteria, and the sources and methods of selection of participants. Describe methods of follow-up  *Case-control study*—Give the eligibility criteria, and the sources and methods of case ascertainment and control selection. Give the rationale for the choice of cases and controls  *Cross-sectional study*—Give the eligibility criteria, and the sources and methods of selection of participants | 9-10 |
|  |  | (*b*) *Cohort study*—For matched studies, give matching criteria and number of exposed and unexposed  *Case-control study*—For matched studies, give matching criteria and the number of controls per case | - |
| Variables | 7 | Clearly define all outcomes, exposures, predictors, potential confounders, and effect modifiers. Give diagnostic criteria, if applicable | 10-11 |
| Data sources/ measurement | 8* | For each variable of interest, give sources of data and details of methods of assessment (measurement). Describe comparability of assessment methods if there is more than one group | 10-11 |
| Bias | 9 | Describe any efforts to address potential sources of bias | 11 |
| Study size | 10 | Explain how the study size was arrived at | 10 |

Continued on next page

| Quantitative variables | 11 | Explain how quantitative variables were handled in the analyses. If applicable, describe which groupings were chosen and why | 10-13 |
| --- | --- | --- | --- |
| Statistical methods | 12 | (*a*) Describe all statistical methods, including those used to control for confounding | 11-13 |
|  |  | (*b*) Describe any methods used to examine subgroups and interactions | 13 |
|  |  | (*c*) Explain how missing data were addressed | 10 |
|  |  | (*d*) *Cohort study*—If applicable, explain how loss to follow-up was addressed  *Case-control study*—If applicable, explain how matching of cases and controls was addressed  *Cross-sectional study*—If applicable, describe analytical methods taking account of sampling strategy | - |
|  |  | (*e*) Describe any sensitivity analyses | 18 |
| Participants | 13* | (a) Report numbers of individuals at each stage of study—eg numbers potentially eligible, examined for eligibility, confirmed eligible, included in the study, completing follow-up, and analysed | 13-14 |
|  |  | (b) Give reasons for non-participation at each stage | - |
|  |  | (c) Consider use of a flow diagram | - |
| Descriptive data | 14* | (a) Give characteristics of study participants (eg demographic, clinical, social) and information on exposures and potential confounders | 13-14 |
|  |  | (b) Indicate number of participants with missing data for each variable of interest | 10 |
|  |  | (c) *Cohort study*—Summarise follow-up time (eg, average and total amount) | - |
| Outcome data | 15* | *Cohort study*—Report numbers of outcome events or summary measures over time | 13-18 |
|  |  | *Case-control study—*Report numbers in each exposure category, or summary measures of exposure |  |
|  |  | *Cross-sectional study—*Report numbers of outcome events or summary measures |  |
| Main results | 16 | (*a*) Give unadjusted estimates and, if applicable, confounder-adjusted estimates and their precision (eg, 95% confidence interval). Make clear which confounders were adjusted for and why they were included | 13-14 |
|  |  | (*b*) Report category boundaries when continuous variables were categorized | - |
|  |  | (*c*) If relevant, consider translating estimates of relative risk into absolute risk for a meaningful time period | - |

Continued on next page

| Other analyses | 17 | Report other analyses done—eg analyses of subgroups and interactions, and sensitivity analyses | 14-19 |
| --- | --- | --- | --- |
| Key results | 18 | Summarise key results with reference to study objectives | 19 |
| Limitations | 19 | Discuss limitations of the study, taking into account sources of potential bias or imprecision. Discuss both direction and magnitude of any potential bias | 22-23 |
| Interpretation | 20 | Give a cautious overall interpretation of results considering objectives, limitations, multiplicity of analyses, results from similar studies, and other relevant evidence | 22-23 |
| Generalisability | 21 | Discuss the generalisability (external validity) of the study results | 22-23 |
| Other information | |
| Funding | 22 | Give the source of funding and the role of the funders for the present study and, if applicable, for the original study on which the present article is based | 29 |
